# Clinical confirmation of an infection with *Echinococcus multilocularis* (Mongolian genotype): first case report of human alveolar echinococcosis in Inner Mongolia, China

**DOI:** 10.1186/s40249-025-01342-4

**Published:** 2025-07-24

**Authors:** Xu Wang, Zhan-Jun Xiao, Chui-Zhao Xue, Wen-Ting Wu, Jiang-Hui Yang, Chun Yan, Ying Wang, Yan Kui, Wen-Bo Luo, Xi Du, Run-Na Zan, Rong-Jian Shang, Sa Li, Rigen Na, Shuai Han, Shi-Zhu Li

**Affiliations:** 1 National Key Laboratory of Intelligent Tracking and Forecasting for Infectious Diseases, National Institute of Parasitic Diseases, Chinese Center for Disease Control and Prevention; Chinese Center for Tropical Diseases Research; Key Laboratory On Parasite and Vector Biology, Ministry of Health; World Health Organization Collaborating Centre for Tropical Diseases; National Center for International Research on Tropical Diseases, Ministry of Science and Technology, Shanghai, China; 2 Xilingol League Central Hospital, Xilinhot, China; 3 Beijing Center for Disease Control and Prevention, Beijing, China; 4 Beijing Tsinghua Changgung Hospital, Beijing, China; 5 New Barga Right Banner Center for Disease Control and Prevention, Hulun-Buir, China; 6 Inner Mongolia Autonomous Regional Center for Disease Control and Prevention, Hohhot, China; 7 Chifeng City Center for Disease Control and Prevention, Chifeng, China; 8 Bairin Left Banner Center for Disease Control and Prevention, Chifeng, China; 9 Changping District Center for Disease Control and Prevention, Beijing, China

**Keywords:** Alveolar echinococcosis, *Echinococcus multilocularis*, Mongolian genotype, Inner Mongolia, China

## Abstract

**Background:**

Alveolar echinococcosis (AE), caused by the larval stage of *Echinococcus multilocularis*, poses a substantial global health challenge due to its high mortality profile. This study reports the inaugural human infection of echinococcosis caused by the Mongolian genotype of *E. multilocularis* in China, also the first reported indigenous AE case in Inner Mongolia.

**Case presentation:**

A 58-year-old female pastoralist from Inner Mongolia, who had no endemic region exposure history but prolonged occupational contact with dogs, presented with severe AE. Clinical examinations revealed a massive hepatic lesion exceeding 10 cm in diameter, accompanied by elevated eosinophils (0.90 × 10^9^/L) and basophils (0.08 × 10^9^/L). Despite undergoing liver transplantation, the patient succumbed postoperatively. Histopathological confirmation and molecular phylogenetics identified the Mongolian genotype of *E. multilocularis* infection, distinct from the predominant Asian genotype in China. Potential evidence of zoonotic transmission was discovered through genotype-matched *E. multilocularis* detection in corsac fox (*Vulpes corsac*) feces from the grasslands along the shores of Hulun Lake (Hulun Buir City, northeastern Inner Mongolia, China).

**Conclusions:**

This report provides the primary evidence of a locally acquired human AE infection in China caused by the Mongolian genotype of *Echinococcus multilocularis*. The discovery of this case challenges historical classifications of echinococcosis endemic areas. The findings call for revised AE-endemic identification criteria, improved AE diagnostic protocols, and enhanced AE surveillance in the Inner Mongolia region to generate further epidemiological evidence and information on disease progression.

## Background

Alveolar echinococcosis (AE), a life-threatening zoonotic disease caused by the infiltrative proliferation of *Echinococcus multilocularis* larvae, has emerged as a critical public health challenge due to its complex pathogenesis and dismal prognosis. Characterized by tumor-like hepatic lesions resembling malignancies or abscesses, AE progresses insidiously, with untreated cases exhibiting a staggering 5-year mortality rate exceeding 90% [1]. The World Health Organization categorizes AE as a neglected tropical disease, yet its endemicity spans the Northern Hemisphere, including Asia, Europe, and North America, predominantly in rural and pastoral communities, with an estimated global annual incidence of 10,489 cases [2]. Transmission occurs through a complex sylvatic cycle: definitive hosts (primarily foxes and dogs) shed infective eggs in their feces, contaminating vegetation consumed by intermediate hosts such as rodents. Humans become accidental hosts through ingestion of eggs via contaminated food, water, or direct contact with infected canids [3]. The AE virulence stems from the larvae’s metastatic growth pattern, which progressively destroys hepatic parenchyma and may disseminate to distant organs including lungs and brain, culminating in fatal liver failure if untreated [4]. Despite advances in antiparasitic chemotherapy (e.g., albendazole) and surgical interventions, therapeutic efficacy remains suboptimal due to delayed diagnosis of AE and potential infiltration and spread of *E. multilocularis* in advanced stages [5].

Molecular epidemiological studies have revealed substantial genetic heterogeneity within *E. multilocularis*, with four distinct genotypes shaping global transmission dynamics [6]. The ​​European genotype​​ dominates central and eastern Europe, while the ​​Asian genotype​​ prevails across western China and Central Asia, collectively accounting for the majority of human infections worldwide [7]. Contrastingly, the ​​North American genotype​​, restricted to arctic and subarctic regions (such as North Asia, Northern Europe, and North America), demonstrates attenuated pathogenicity, with minimal human case reports [8]. Of particular scientific interest is the ​​Mongolian genotype​​, which exhibits high genetic variability and a geographically restricted distribution across the Mongolian Plateau and adjacent Russian territories [6, 8]. Current knowledge gaps persist regarding its human infectivity, clinical progression patterns, and responsiveness to standard therapies, largely due to the paucity of confirmed human cases and comprehensive whole-genome sequencing data from endemic regions [7].

China shoulders the greatest global burden of AE, with surveillance data revealing an annual human incidence rate of 0.44 per million population and a 1.30% prevalence in small mammals (predominantly rodents) [9]. The Qinghai-Tibet Plateau constitutes the epicenter of Chinese AE endemicity, reporting an extraordinary annual incidence of 46.95 per million people, over 100-fold higher than the national average [9], attributable to synergistic ecological drivers: high-altitude meadows supporting dense populations of rodents (e.g., voles and pika) and their predators (e.g., foxes), free-roaming dogs bridging wild environments and human communities, and low-temperature climatic conditions favoring *E. multilocularis* egg survival [10, 11]. Paradoxically, ​​Inner Mongolia Autonomous Region​​, despite sharing comparable ecological prerequisites for transmission—vast grasslands, abundant wildlife, prevalent semi-nomadic pastoralism, and a high-latitude climate—remained conspicuously absent from national AE case reports until this study [12]. China’s official criteria for designating echinococcosis endemic areas require both verified transmission cycles between definitive hosts (e.g., canids) and intermediate hosts (e.g., rodents) and documented human infections, a threshold unmet in Inner Mongolia since the establishment of the National Health Information System in 2004 [13, 14]. However, the prior classification of Inner Mongolia as a non-AE-endemic region is now challenged by the detection of *E. multilocularis* in local wildlife and an indigenous human AE case. This study reports the first molecularly confirmed AE case in Inner Mongolia, combining phylogenetic analysis, clinical imaging, histopathological findings, and wildlife infections from the region, aims to provide novel epidemiological insights into AE and advance biological understanding of *E. multilocularis*.

## Case presentation

### Patient history and environmental exposure

In May 2024, a 58-year-old female pastoralist from Bairin Left Banner, Chifeng City, Inner Mongolia, was initially diagnosed with clinical AE by B-mode ultrasound and computed tomography (CT) examinations after enduring intermittent abdominal pain and bloating for approximately one month at Xilingol League Central Hospital [13]. The patient had no lifetime history of internatzional travel, or exposure records to AE endemic areas identified in China (including Sichuan, Xizang, Gansu, Qinghai, Ningxia and Xinjiang) [9], eliminating possibilities of imported infection. Her lifestyle was closely associated with livestock farming, with her family owning more than 200 sheep and seven domestic dogs. The family’s inherent pastures, characterized by semi-arid meadow grassland, served as a mixed habitat for various wildlife, including corsac foxes (*Vulpes corsac*), red foxes (*Vulpes vulpes*), wolves (*Canis lupus*), Brandt’s voles (*Lasiopodomys brandtii*), and Daurian ground squirrels (*Spermophilus dauricus*) [15], all potential participants in the sylvatic cycle of *E. multilocularis*.

### Clinical examination and diagnosis

**Fig. 1:**
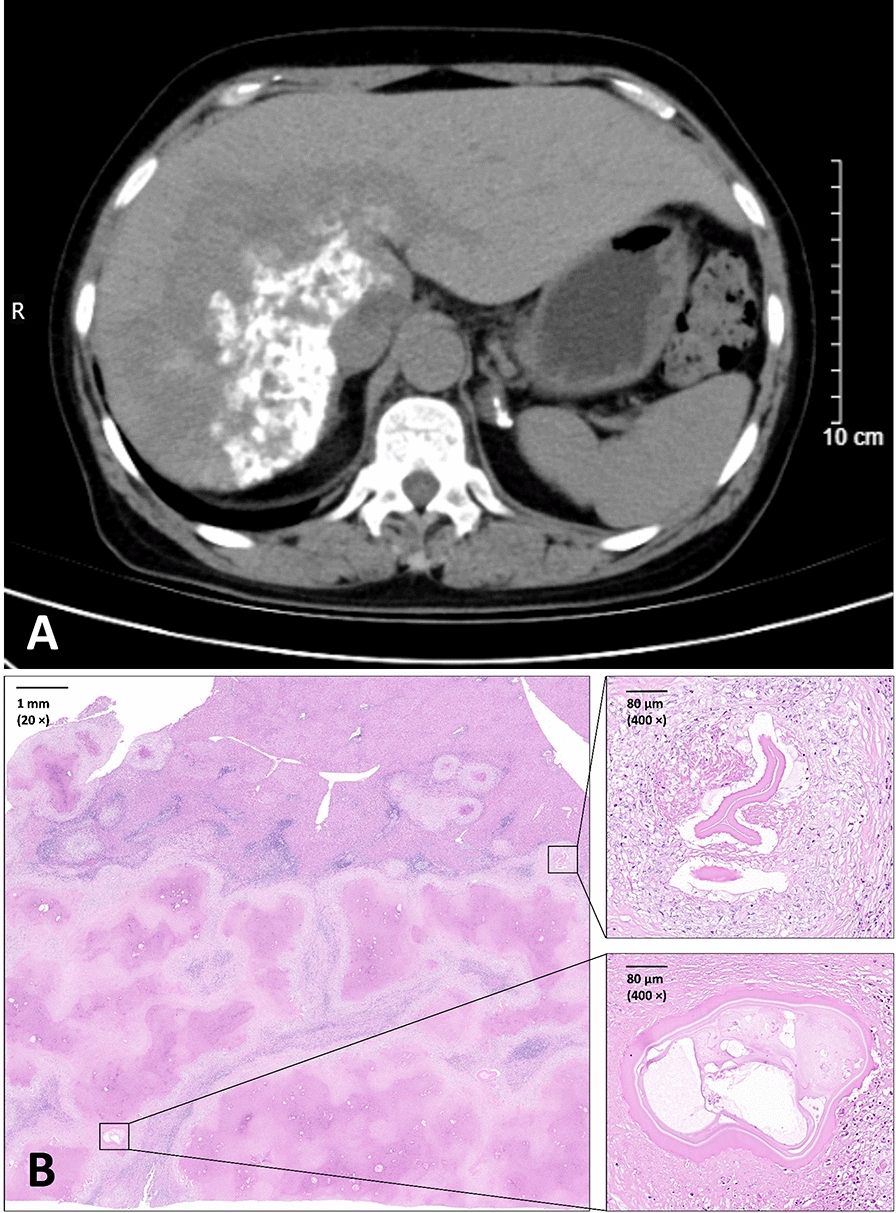
Computed tomography images and pathological observations of lesion

In May 2024, the patient underwent comprehensive evaluation and treatment at Beijing Tsinghua Changgung Hospital. Routine blood examination revealed elevated eosinophils (0.90 × 10^9^/L) and basophils (0.08 × 10^9^/L), suggesting a helminthic infection. Liver function tests showed elevated alkaline phosphatase (179 U/L) and γ-glutamyl transferase (58 U/L), indicating cholestatic injury. D-dimer levels were mildly elevated (0.87 mg/L FEU), reflecting possible thromboembolic complications (Table 1). Abdominal CT revealed a massive, infiltrative hepatic lesion (79 × 136 × 123 mm), mainly in the right lobe, involving the caudate lobe (segment S1) and the left lateral (S2) and medial (S4) lobe. The lesion exhibited blurred boundaries and patchy dense shadows visible inside (Fig. 1A). These features are consistent with AE but also similar to hepatocellular carcinoma or hepatic abscess.

**Table 1:** Abnormal results of the patient’s preoperative examination

Codes	Description	Results	Reference range	Units of measurement
EOS#	Eosinophil count	0.90 ↑	0.02‒0.52	10^9^/L
BASO#	Basophil count	0.08 ↑	0.00‒0.06	10^9^/L
EOS%	Eosinophil ratio	14.40 ↑	0.40‒8.00	%
BASO%	Basophil ratio	1.30 ↑	0‒1.00	%
ALP	Alkaline phosphatase	179 ↑	< 135	U/L
γ-GT	γ- glutamyl transferase	58 ↑	Female: 7‒45	U/L
D-dimer	D-dimer	0.87 ↑	0‒0.55	mg/L FEU

### Surgical intervention and outcome

In June 2024, liver transplantation surgery was performed in an attempt to treat the patient, reflecting the lesion’s unresectability due to the severity of the infection and the unpredictable risk of recurrence. Despite meticulous surgical technique and postoperative immunosuppression management, the patient succumbed within a few days post-operation, a fatal outcome underscoring lethality in advanced stages of AE. Histopathological analysis of explanted liver tissue via hematoxylin–eosin staining indicated the absence of *Echinococcus* protoscoleces or hooks, but revealed germinal layer structures on irregular vesicle walls in the lesion tissue, along with hyperplasia of fibrous tissue outside the lesions (Fig. 1B). The identification of germinal layer remnants provided evidence of *E. multilocularis* infection. However, these clinical and pathological features did not exhibit significant peculiarity when compared with those of other AE cases.

### Pathological and molecular confirmation

**Fig. 2:**
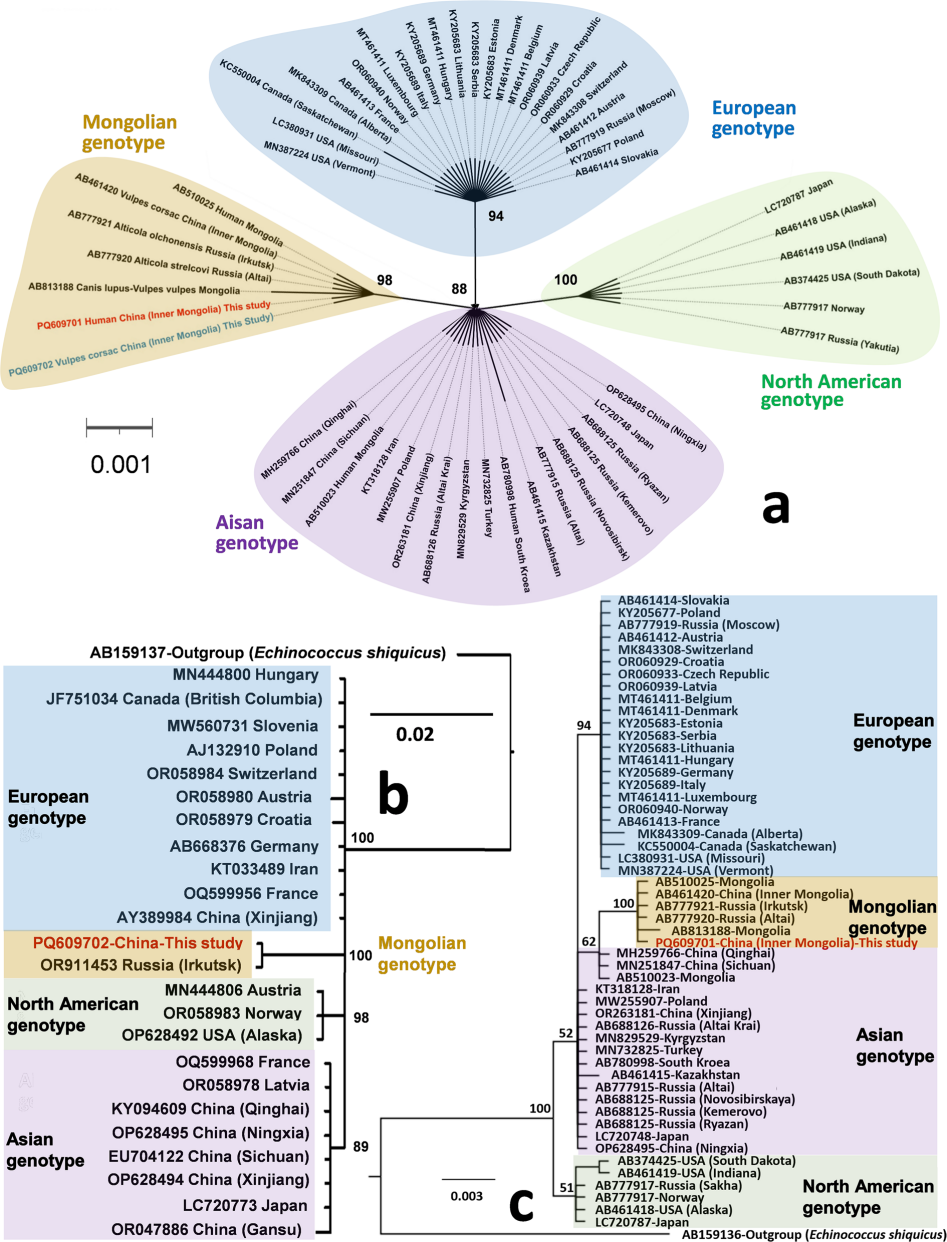
Phylogenetic tree of Bayesian inference based on partial *cox1* and *nad1* genes. **a** Bayesian phylogenetic analysis based on 243-bp *cox1* gene and “HKY + F” substitution models setting; **b** analysis based on 471-bp *cox1* gene and “HKY + I”; **c**. analysis based on 529-bp *nad1* gene and “HKY + F + I”

To resolve etiological uncertainties, total DNA was extracted from formalin-fixed and paraffin-embedded (FFPE) lesion specimens using QIAamp Kits (Qiagen, Hilden, Germany) numbered 56404 [16]. Targeted amplification of mitochondrial markers, a 471-bp fragment of the cytochrome c oxidase subunit I (*cox*1) gene and a 529-bp fragment of the nicotinamide dehydrogenase subunit I (*nad*1) gene, were amplified via polymerase chain reaction (PCR) using JB3/4.5 (F/5′-TTTTTTGGGCATCCTGAGGTTTAT-3′ and R/5′-TAAAGAAAGAACATAATGAAAATG-3′, with an annealing temperature of 55 °C) and JB11/12 (F/5′-AGATTCGTAAGGGGCCTAATA-3′ and R/5′-ACCACTAACTAATTCACTTTC-3′, 50 °C) primers, respectively [17, 18]. The PCR products were sequenced at Sangon Biotech (Shanghai) Co., Ltd. (Shanghai, China). The obtained *cox*1 and *nad*1 sequences were aligned with existing sequences in the NCBI database, revealing highest similarities (100.00% with 100% coverage and 99.59% with 100% coverage, respectively) to sequences under accession numbers AB777921 (Mongolian Genotype) and OR911453 (Mongolian Genotype). The resulting sequences have been deposited in the GenBank database under accession numbers PQ609701 and PQ609666, respectively. Then, a total of 77 globally representative sequences (53 for *cox*1 gene and 25 for *nad*1 gene), including sequences obtained in this case and retrieved from GenBank, were used for Bayesian phylogenetic analysis by MrBayes 3.2.4 (http://nbisweden.github.io/MrBayes/index.html) [19]. The *nad1* (Fig. 2b) and *cox1* (Fig. 2c) phylogenies positioned the strain from the patient as a sister branch to isolates from Mongolia and Russia in the Mongolian genotype group, with strong nodal support (posterior probability = 1.00).

### Epidemiological surveys for animal hosts

In September 2024, multi-sectoral teams conducted an extensive epidemiological field surveys across Chifeng and Hulun-Buir Cities in Inner Mongolia, to investigate the prevalence of *E. multilocularis* in animals. These two localities share similar ecological settings, including the Mongolian Plateau, the edge of the Gobi Desert, steppe habitats, and the presence of the *V. corsac* and Brandt’s vole (*Lasiopodomys brandtii*). A total of 270 carnivore fecal samples (225 from dogs, 44 from foxes, and 1 from a cat) and 171 liver samples from rodents (89 *L. brandtii*, 77 *Rattus norvegicus*, 3 *Spermophilus dauricus*, 1 *Cricetulus barabensis*, and 1 *Phodopus roborovskii*) were collected. DNA extractions were performed using QIAamp Kits (Qiagen, Hilden, Germany) numbered 51404 and 51604 for ethanol-preserved liver samples and −80 °C-inactivated feces, respectively. A 243-bp *cox*1 gene was amplified by nested PCR using first (exF/5′-TTGAATTTGCCACGTTTGAATGC-3′ and exR/5′-GAACCTAACGACATAACATAATGA-3′) and second (Em-inF/5′-GTCATATTTGTTTAAGTATAAGTGG-3′ and Em-inR/5′-CACTCTTATTTACACTAGAATTAAG-3′) round primers with the annealing temperature of 52 °C [20]. Phylogenetic analysis of sequencing results showed that a *cox1* fragment of the Mongolian genotype of *E. multilocularis* was detected in a fecal sample of *V. corsac* from the shore of Hulun Lake in New Barga Right Banner, Hulun-Buir City (Fig. 2a), whereas all other samples were negative. The sequence is registered under the accession number PQ609702 in NCBI.

## Discussion

### Taxonomic classification and distribution of the Mongolian genotype

**Fig. 3:**
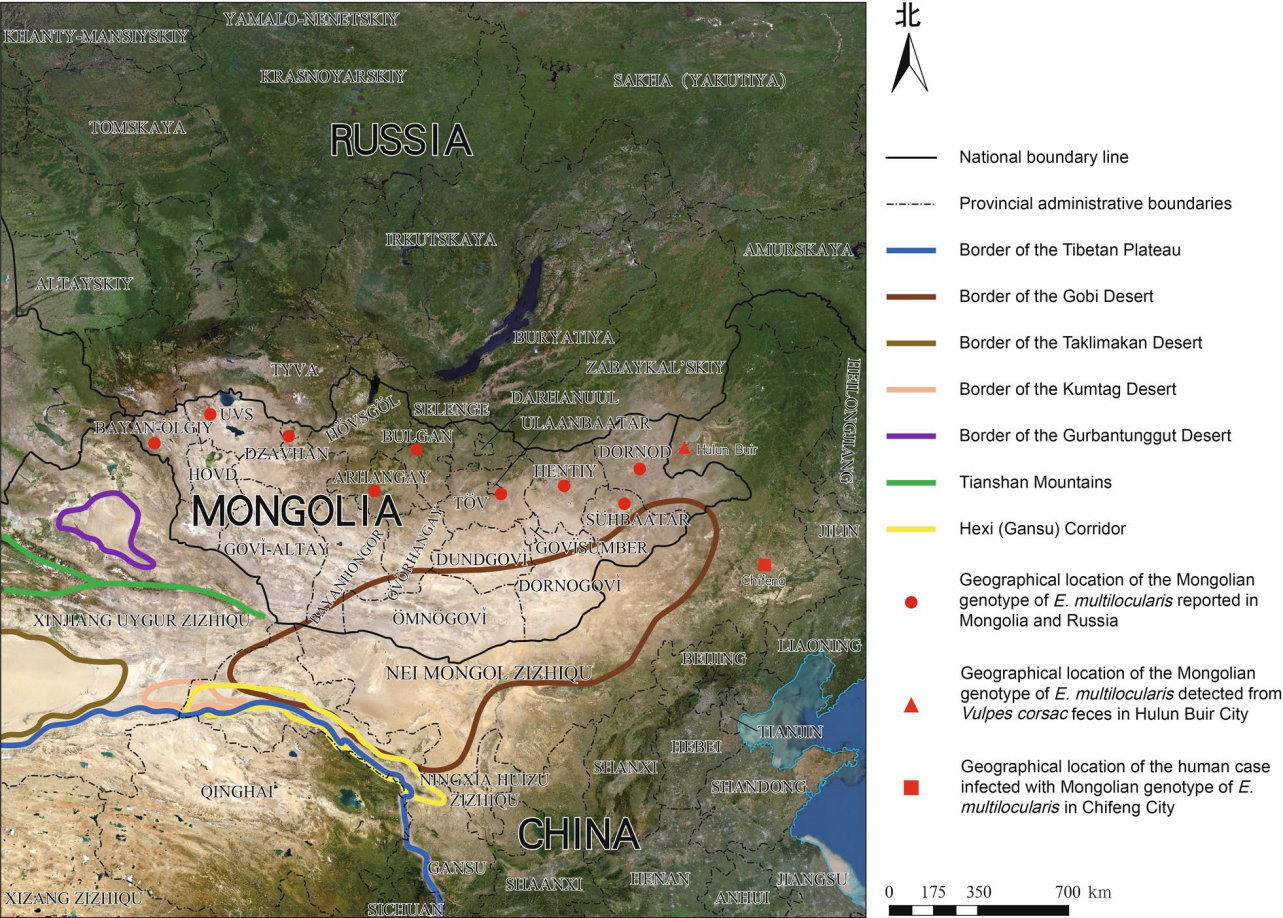
Distribution map of Mongolian *Echinococcus multilocularis* reported globally. Map approval No.: GS (2005) 2043

The taxonomic status of the Mongolian genotype of *E. multilocularis* has undergone significant revisions since its initial discovery. In 2007, Tang et al*.* [21] proposed *Echinococcus russicensis* as a novel species based on morphology of the adult from *Echinococcus* strains, isolated from *V. corsac* in the northward Greater Khingan Range of Hulun-Buir City. However, subsequent multi-locus analyses incorporating mitochondrial (cytochrome *b*, NADH dehydrogenase subunit II, *cox1*) and nuclear (elastin-like polypeptide) genes by Nakao et al*.* [6] in 2009 suggested that despite its high genetic variation rate, this strain could still be considered an intra-specific variant, the Mongolian genotype, within *E. multilocularis*. Current biogeographic data delineate its distribution across 10 Mongolian provinces (Arkhangai, Bulgan, Dornod, Zavkhan, Khentii, Sukhbaatar, Tuv, Uvs, Bayan-Ulgii, and Ulaanbaatar) and two Russian regions (Irkutsk Oblast and Altai Republic) [22–24] (Fig. 3), forming a discontinuous arc along the steppe vegetation belt on the northern and eastern edge of the Gobi Desert on the Mongolian Plateau [25]. This restricted distribution and distinct genetic characteristics reflect a complex interplay of ecological specialization and historical biogeography: (1) Co-maintenance of key native definitive host (*V. corsac*) and intermediate host (*Alticola* spp.) adapted to xeric conditions; (2) Quaternary climate oscillations (last glacial period) that isolated ancestral populations in Pleistocene grassland refugia [6]; (3) Geographical barriers including the Hexi (Gansu) Corridor, Gobi Desert and Tianshan Mountains, which impede host migration and parasite’s gene flow between Mongolian Steppe and heavily AE endemic areas of Tibetan Plateau and Central Asia (Fig. 3).

### Pathogenicity and clinical significance

Previously, only two human infections of the Mongolian genotype have been reported globally, both occurring in Mongolia. The first case involved a 25-year-old male born in Uvs Province who was diagnosed with a hepatic malignancy featuring a 15 × 9.5 cm lesion in the right liver in 2006. The histopathological confirmation of AE was obtained from the resected lesion of the patient who died of liver failure 5 days after the lesion resection surgery. The second case concerned a 20-year-old female from Bayan-Ulgii Province diagnosed in 2009 with AE manifesting as a 6.3 cm × 6.2 cm right hepatic lesion, but her postoperative outcomes remain undocumented [22]. These limited clinical data reveal no distinctive clinical manifestations, blood test findings, imaging characteristics, or pathological features specifically attributable to AE caused by Mongolian genotype. Therefore, further comparative clinical studies, including confirmed Mongolian genotype infections, are required to investigate potential genotype-specific disease expression. In addition, current epidemiological data restrict confirmed hosts of this genotype to wildlife species: definitive hosts comprise *V. corsac*, *V. vulpes*, and *C. lupus*, intermediate hosts include *L. brandtii*, *Alticola strelzowi*, and *A. olchonensis* (Figure S2) [21, 23, 24]. The genotype’s compatibility with dogs remains unverified, which is a critical knowledge gap given dogs’ established role as zoonotic bridges in *E. multilocularis* transmission cycles [10]. This potential host restriction could partially explain the genotype’s sparse human case reports. Comprehensive characterization of Mongolian genotype pathogenicity in human and animal hosts represents an urgent research priority for improving the diagnosis, treatment and surveillance of AE cases caused by this genotype. Such understanding will inform targeted control strategies, particularly regarding human exposure prevention in endemic zones and regions of elevated transmission risk.

### Epidemiological implications

The detection of the inaugural alveolar echinococcosis (AE) case attributed to the Mongolian genotype in China carries substantial epidemiological significance. Inner Mongolia’s geographical contiguity with Mongolia, a region demonstrating elevated Mongolian genotype prevalence, heightens concerns regarding transboundary AE transmission through wildlife reservoirs. Cross-border migration of wild canids, particularly foxes, may serves as a potential pathway for *E. multilocularis* spread. This finding further challenges prior epidemiological classifications designating Inner Mongolia as a non-endemic AE area [12]. Consequently, enhanced molecular epidemiological surveillance​ (including wildlife, domestic dogs, and even humans) for AE in Inner Mongolia is imperative ​​to track Mongolian genotype distribution patterns,, and to enable a more accurate assessment of the true AE prevalence within the region. Critical intervention strategies must encompass specialized AE diagnostic training for medical professionals in Inner Mongolia, followed by retrospective reviews of liver malignancy diagnoses to identify potentially misclassified AE cases.​ Furthermore, annual epidemiological surveillance should be strengthened to monitor the transmission dynamic of the Mongolian genotype. Such longitudinal data will enable public health authorities to institute preemptive containment measures, including targeted control of definitive and intermediate hosts [9].

### Additional insights

The emergence of Mongolian genotype-associated human infection in Inner Mongolia likely stems from three synergistic factors: anthropogenic activities, ecosystem dynamics, and climatic shifts. Traditional pastoral practices involving dog guardianship of livestock, coupled with allowing them to prey on wildlife, sustain the natural spread of parasites and spillover risks to humans [10]. Uncontrolled wildlife movement across the China-Mongolia frontier facilitates the novel parasite strains into previously unaffected ecosystems. Concurrently, climate-mediated environmental modifications, particularly temperature elevation and grassland rehabilitation in northern China, have precipitated wildlife range expansion and intensified human-wildlife interface [26]. These intersecting drivers underscore the potential utility of the EcoHealth paradigm, which integrates ecological, social, and health system dimensions, as a viable framework for addressing these interconnected challenges [27].

### Limitations

DNA extraction from formalin-fixed paraffin-embedded (FFPE) tissues encountered substantial technical constraints. Formalin-induced DNA damage precluded full renaturation during extraction, rendering amplification of long sequences (e.g., complete 1608-bp *cox1* and 894-bp *nad1* genes) infeasible. The 471-bp *cox1* and 529-bp *nad1* gene fragments obtained in this study currently represent the longest DNA sequences retrievable from the available samples. Notably, the primers for these sequences were originally designed by Bowles et al. for *Echinococcus* genotype classification, ensuring reliable genotyping [17, 18]. Parallel challenges emerged in definitive host fecal DNA extraction, requiring precise disruption of embryophore membranes without compromising DNA integrity. Extraction efficacy correlated with egg burden, with many samples below amplification thresholds. High-sensitivity nested PCR successfully amplified one sample, though conventional PCR failed for longer fragments. Phylogenetic analysis confirmed genotyping reliability despite fragment length disparities: the 244-bp cox1 sequence (Fig. 2a) exhibited congruent clustering patterns with its 471-bp counterpart (Fig. 2b), validating this fragment’s utility for *E. multilocularis* genotyping. Furthermore, the linear spatial distance exceeding 500 km between Chifeng City (the patient’s location) and Hulunbuir City (the *V. corsac* infection site) diminishes the evidence for direct zoonotic transmission. However, these two localities possess comparable climatic and ecological profiles (including vegetation and wildlife), and similar livestock production practices, thus demonstrating the potential risk of zoonotic exposure.

## Conclusions

This paper reports the first documented AE case caused by the Mongolian genotype of *E. multilocularis* in China. The patient’s lethal outcome underscores the pathogenic severity of this genotype, with significant implications for understanding AE epidemiology in China, especially in regions adjacent to Mongolia. Nevertheless, definitive evidence of established local transmission cycles involving competent intermediate hosts in Inner Mongolia remains limited.

​​Consequently, a comprehensive reassessment of AE diagnostic protocols and preventive measures in Inner Mongolia is urgently required. Priority actions include retrospective analysis of hepatic malignancy diagnoses and revision of AE-endemic district designations, in addition to enhanced surveillance for echinococcosis in humans and animals in Inner Mongolia and other border regions to contain the potential transmission of emerging *Echinococcus* strains.

From a global perspective, more research is required on the genome, pathogenesis, and epidemiology of the Mongolian genotype. This will not only improve our understanding of the disease but also help in developing more effective treatment and prevention strategies for AE caused by this genotype. In addition, based on the One Health approach, international cooperation is essential, especially between China and Mongolia, to share information and resources for better control of this emerging infectious disease. By taking these steps, we can hope to reduce the burden of AE caused by the Mongolian genotype and improve the health outcomes of affected individuals.

## Data Availability

All data generated or analysed during this study are included in this published article.

## Abbreviations

AE: Alveolar echinococcosis
CT: Computed tomography
FFPE: Formalin-fixed paraffin-embedded
PCR: Polymerase chain reaction
*cox*1: Cytochrome c oxidase subunit I
*nad*1: Nicotinamide dehydrogenase subunit I
